# A new synthetic protein, TAT-RH, inhibits tumor growth through the regulation of NFκB activity

**DOI:** 10.1186/1476-4598-8-97

**Published:** 2009-11-09

**Authors:** Daniela Sorriento, Alfonso Campanile, Gaetano Santulli, Eleonora Leggiero, Lucio Pastore, Bruno Trimarco, Guido Iaccarino

**Affiliations:** 1 Dipartimento di Medicina Clinica, Scienze Cardiovascolari ed Immunologiche, Università Federico II, Napoli, Italia; 2 CEINGE-Biotecnologie Avanzate, Napoli, Italia; 3 Dipartimento di Biochimica e Biotecnologie Mediche, Università degli Studi di Napoli "Federico II", Napoli, Italia

## Abstract

**Background:**

Based on its role in angiogenesis and apoptosis, the inhibition of NFκB activity is considered an effective treatment for cancer, hampered by the lack of selective and safe inhibitors. We recently demonstrated that the RH domain of GRK5 (GRK5-RH) inhibits NFκB, thus we evaluated its effects on cancer growth.

**Methods:**

The role of GRK5-RH on tumor growth was assessed in a human cancer cell line (KAT-4). RH overexpression was induced by adenovirus mediated gene transfer; alternatively we administered a synthetic protein reproducing the RH domain of GRK5 (TAT-RH), actively transported into the cells.

**Results:**

*In vitro*, adenovirus mediated GRK5-RH overexpression (AdGRK5-NT) in human tumor cells (KAT-4) induces IκB accumulation and inhibits NFκB transcriptional activity leading to apoptotic events. In BALB/c nude mice harboring KAT-4 induced neoplasias, intra-tumor delivery of AdGRK5-NT reduces in a dose-dependent fashion tumor growth, with the highest doses completely inhibiting it. This phenomenon is paralleled by a decrease of NFκB activity, an increase of IκB levels and apoptotic events. To move towards a pharmacological setup, we synthesized the TAT-RH protein. In cultured KAT-4 cells, different dosages of TAT-RH reduced cell survival and increased apoptosis. In BALB/c mice, the anti-proliferative effects of TAT-RH appear to be dose-dependent and highest dose completely inhibits tumor growth.

**Conclusion:**

Our data suggest that GRK5-RH inhibition of NFκB is a novel and effective anti-tumoral strategy and TAT-RH could be an useful tool in the fighting of cancer.

## Background

NFκB is a dimeric, ubiquitous transcription factor involved in cellular responses to stimuli such as stress, cytokines, free radicals, ultraviolet irradiation, oxidized LDL, and bacterial or viral antigens [1-5]. In basal conditions, NFκB dimers are sequestered in the cytoplasm by a family of inhibitors, called IκBs, that bind NFκB by means of ankyrin repeat domains masking its nuclear localization signals (NLS); such interaction blocks NFκB in an inactive form in the cytoplasm [6]. Activation of NFκB is initiated by the signal-induced phosphorylation of IκB proteins by IκB kinase (IKK), thus inducing IκB ubiquitination and degradation by the proteasome. At this time, NFκB is active and stably localized within the nucleus where it induces the expression of specific genes. The activation of these genes by NFκB then leads to inflammation, immune response, cell survival, or cellular proliferation depending on cell type. The pathogenetic role of NFκB has been clarified in many diseases [7-9], such as type II diabetes and insulin-resistance [10,11]; cardiac hypertrophy [12]; atherosclerosis [13]; chronic heart failure [14]; cancer and angiogenesis [15].

There are many evidences about the role of NFκB in cancer. Indeed, Hanahan and Weinberg identified the six hallmarks that characterized tumor cells (Self-Sufficiency in Growth Signals, Insensitivity to Anti-growth Signals, Evading Apoptosis, Limitless Replicative Potential Sustained Angiogenesis, Tissue Invasion and Metastasis) [16,17] and most of the genes that regulate such effects are under the transcriptional control of NFκB. Moreover, NFκB transcription activity is constitutively increased in many tumors like melanoma [18,19], thyroid [20,21] and colon [22] carcinoma. The mechanisms determining persistent and de-regulated NFκB activity in cancer cells are not well understood but a major role is probably played by the cellular concentration of the inhibitory protein IκB. In particular, the constitutive activation of NFκB in solid tumors has been mainly attributed to decreased IκB levels due to defective IκBα activity, constitutive IKK activity, enhanced proteasome activity, etc.

To date, different approaches have been developed to block NFκB in several conditions. A successful one is using a proteasome inhibitor, PS-341, to treat patients with refractory or resistant multiple myeloma [23]. A protein that disrupts the association of the IKK complex is used to prevent inflammatory bone destruction [24]. The inhibition of IκBα phosphorylation by the Bay 11-7082 compound, has been successfully used to prevent tumor growth and leukemic infiltration in a mouse model of adult T cell leukemia [25]. Furthermore, inhibition of NFκB activation by expression of a mutant IκBα, which is resistant to phosphorylation and degradation, increased NFκB dependent apoptosis to stimuli such as TNFα [26,27]. All these approaches open new fields for the management of NFκB-associated diseases like cancer.

G protein coupled receptor (GPCR) kinases (GRKs) regulates GPCRs signaling by inducing receptor desensitization. Recent findings unveil new cellular function for these kinases. Indeed, we have recently demonstrated that GRK5 regulates the activity of the transcription factor NFκB [28]. In particular, in endothelial cells GRK5 is able to bind the inhibitory protein of NFκB, IκBα, by means of the RH domain (GRK5-RH) and stabilize the complex IκBα/NFκB in the nucleus, thus inhibiting NFκB transcriptional activity [28]. Indeed, RH overexpression, which causes GRK5-IκBα interaction, inhibits the transcriptional activity and DNA binding of NFκB both in basal condition and after stimulation with LPS.

Given the notion that the modulation of NFκB transcriptional activity is an effective therapeutic strategy for cancer, we hypothesized that RH, being a potent inhibitor of NFκB, could reveal therapeutic potentialities in oncology. We therefore tested in a human tumor cell line, KAT-4, the effects of GRK5-RH on cell growth either in cultured cells or in tumors in BALB/c nude mice, grown after subcutaneous injection of KAT-4 cells.

## Materials and methods

### Cell culture

Human tumor cells (KAT-4) were a kind gift of Dr. Maddalena Illario (Federico II University, Dept. of Cellular and Molecular Pathology). This cell line has been recently authenticated by short tandem repeat (STR) profiling which demonstrated, despite the widespread knowledge, that KAT-4 are not of thyroid cancer origin but share the same STR profile with the HT-29 colon cancer cell line [29]. Cells were cultured in Dulbecco's minimal essential medium (DMEM) supplemented with 10% foetal bovine serum (FBS) at 37°C in 95% air-5%CO_2_.

### Plasmid constructs

In order to synthesize a protein reproducing the RH domain of GRK5, engineered to be actively transported into the cells by means of TAT sequence, we designed the TAT-RH plasmid, using pcDNA3.1-GRK5-RH [28] as template to amplify RH sequence. The primers were designed with the addition of NCO I and KPN I restriction sites sequences: 5'CCCCCATGGCCCGAGATTACTGCAGTTTA3' and

3'ATAAAACTAGCGAAAGAGATCCCATGGGGG5';

Amplified sequences were purified by gel extraction kit (Invitrogen) and cloned into pTAT-HA vector a kind gift of Dr. Steven Dowdy (Washington University School of Medicine) [30] by means of T4 DNA ligase (Promega). The right frame and orientation were confirmed by restriction analysis and DNA sequencing (Avant 3100, Applied Biosystem). pTAT-HA vector comprises the 11 aminoacid TAT domain, that is the minimum sequence of HIV TAT able to transduce into cells, and histidine and HA as tags.

### Production of adenoviral vectors

The adenoviral construct GRK5-NT (AdGRK5-NT) used for gene transfer *in vivo *has been previously described [28]. AdGRK5-NT comprises the N terminal and RH domains of GRK5 conjugate to Green Fluorescen Protein gene (GFP) under the CMV promoter.

For Lac-z adenoviral construct, we subcloned Lac-Z gene, excised by digestion from pcDNA 3.1/Lac-Z (Invitrogen), into the AscI site of the pShuttle-linker plasmid, and the resulting plasmid was used to generate the first generation of adenoviruses (AdLac-Z).

First-generation of adenoviral vectors AdGRK5-NT and AdLac-Z was amplified in 293N3S cells in monolayer for small-scale amplification and in suspension for large scale amplification. Virus production was performed as follows: 3 liters of 293N3S cells at a concentration of 3-4 × 10^5 ^cells/ml were harvested by centrifugation and resuspended in 5% of the volume of conditioned medium and then infected with all the crude lysate obtained from the infection of two 150-mm dishes. Virus adsorption was performed at 37°C on a magnetic stir plate for 2 hours; after this step, medium (25% conditioned and 75% fresh Joklik's MEM supplemented with 5% FBS) was added to a final volume of 2 liters. Infected cells were harvested after 48 hours, lysed and resuspended in TM solution (10 mM Tris-HCl pH 8.0, 2 mM MgCl_2_). After three freeze-thaw cycles cells were incubated in 2 M MgCl_2 _in presence of DNase (10 mg/ml) for 1 hour at 37°C. After DNAase incubation, cellular debris were eliminated by centrifugation at 3500 rpm for 15 minutes and the remaining lysate was subjected to ultracentrifugation on a continuous CsCl_2 _gradient at 35,000 rpm for 2 hours at 4°C. The upper band, which contains empty particles, was eliminated and a second ultracentrifugation on a continuous CsCl gradient (35,000 rpm for 18 hr at 4°C) was performed. The harvested vector was dialyzed twice against TM with 4% sucrose and stored at -80°C until use. Vector concentration (particle number) was determined by UV spectrophotometric analysis at 260 nm.

### Protein synthesis and purification

TAT and TAT-RH plasmids were transformed into a BL21 (DE3) pLysS (Invitrogen) bacterial strain. 2 L of Luria Broth (LB) culture was grown overnight and then Isopropylthiogalactoside (IPTG, 100 μM, 3 hrs) was added to induce protein expression. For protein purification we used denaturing conditions to recover all the recombinant proteins from bacterial inclusion bodies (Lysis buffer: 8 M Urea, 100 mM NaCl, 20 mM Hepes pH 8). Ni-NTA columns (GE Healthcare) were used for protein purification. Columns were pre-equilibrated with 10 mM imidazole; lysates were clarified by sonication and applied to the columns. After extensive washing with lysis buffer plus 20 mM imidazole, recombinant protein were eluted with increasing amounts of imidazole (100, 200 and 500 mM). Protein refolding was realized by buffer exchanging into Tris 1 M pH 7.5 using Amicon Ultra-4 Centrifugal Filters (Millipore). The recombinant protein TAT-RH was added to the culture medium in KAT-4 cells for the *in vitro *study and injected intra-tumor for the *in vivo *study. TAT protein was used as control.

### Nuclear extracts preparation, Immunoprecipitation and western blot and Apoptosis analysis

The experiments were performed as previously described [28].

### Luciferase assay

Cells were transfected with plasmid expression vectors containing the luciferase reporter gene linked to five repeats of a NFκB binding site (κB-Luc) or β-galactosidase (β-Gal) and infected with AdGRK5-NT. Transient transfection was performed using the Lipofectamine 2000 (Invitrogen) according to manufacturer's instruction. Lysates were analysed using the luciferase assay system with reporter lysis buffer from Promega and measured in a β-counter. Relative luciferase activity was normalized against the co-expressed β-galactosidase activity to overcome variations in transfection efficiency between samples.

### Electrophoretic mobility shift assay (EMSA)

EMSA was performed using nuclear extracts. Double stranded NFκB oligonucleotide (5' AGTTGAGGGGACTTTCCCAGGC 3') was end-labelled using [^32^P]-γ ATP (GE Healthcare) and T4 polynucleotide kinase (Roche). Samples were subject to electrophoresis in 8% non denaturing polyacrilamide gels with 0.5% TBE buffer (0,09 M Tris, 0,09 M boric acid, 0,02 M EDTA). Digitalized gels autoradiographies were then quantified (Image Quant).

### *In vivo *Study Design

Experiments were carried out, in accordance to NIH guidelines for Animal Investigation, in 6-weeks-old BALB/c immunoincompetent nude mice (Charles River), which had access to food and water ad libitum. For tumor formation, a suspension containing 2 × 10^6 ^KAT-4 cells in 200 μl of DMEM were injected subcutaneously in the dorsal side of nude mice. Animals were anesthetized using isofluorane 2%. We used mice that developed tumors of approximately 6 mm in 2 weeks. Mice were divided into 9 groups (5 mice/group) and administered twice a week for 17 days (AdGRK5-NT) or 4 weeks (TAT-RH) with intra-tumor injections of the specific treatment.

In particular, two groups received AdGRK5-NT at either low (10^8 ^pfu/ml) or high dosage (10^11 ^pfu/ml) while another group received the higher dose of AdLACZ and was used as negative control. Three more groups of mice were treated with TAT-RH protein at the dosage of 8, 12 or 16 mg/Kg. These mice were compared to two control groups treated with either saline solution or the pTAT-HA protein lacking the RH sequence. Tumor growth was measured by caliper twice a week and by Ultrasound (VeVo 770, Visualsonics) once a week. At the end of the treatment, mice were sacrificed by cervical dislocation and tumors processed for biochemical or histological analysis. All in vivo experimental protocols were approved by the Federico II University Ethical Committed for Animal Studies.

### Real Time PCR

Total RNA was isolated using Trizol reagent (Invitrogen) and cDNA was synthetized by means of Thermo-Script RT-PCR System (Invitrogen), following the manufacturer instruction. After reverse transcription reaction, real-time quantitative polymerase chain reaction (PCR) was performed with the SYBR Green real time PCR master mix kit (Applied Biosystems).

The reaction was visualized by SYBR Green Analysis (Applied Biosystem) on StepOne instrument (Applied Biosystem).

Primers for cytokines gene analysis were as follows:

TNFα: forward, 5'CCAGGAGAAAGTCAGCCTCCT3'; reverse, 5'CGATAAAGGGGTCAGAGTAAT3'; VEGF: forward, 5'CAGGCTGTCGTAACGATGAA3', reverse 5'TTTCTTGCGCTTTCGTTTTT3', GAPDH: forward, 5'AGTATGTCGTGGAGTCTACT3', reverse 5'TGTGG TCATGAGCCCTTCCAC3'.

All values obtained were normalized to the values obtained with the GAPDH primers. The results are expressed as the relative integrated intensity.

### Histology and Immunocytochemistry

Paraffin embedded sections were processed for the triple layered immunocytochemical peroxidase anti-peroxidase (PAP) method. PCNA (Cell signaling), Cleaved caspase 3 (Cell signalling) and Lectin (Sigma) antiserum were used to analyze cell proliferation, death and neo-angiogenesis, respectively. The peroxidase was revealed in presence of 0,03% hydrogen peroxide and of an electron donor, 2,5% diaminobenzidine, which becomes visible as a brown precipitate. For negative controls, the primary antiserum was omitted. Sections were then viewed with an Eclipse E1000 Fluorescence Microscope (Nikon) and acquired using Sigma Scan Pro software (Jandel). For X-GAL staining and GFP visualization, cryostat sections were incubated with β-GAL solution (PBS, 0.02 mM K_3_Fe(CN)_6_, 0.02 mM K_4_Fe(CN)_6_, 0.02 mM MgCl2, 0.002% NP40, 0.05 mg/ml X-GAL stain) for 90 min at 37°C and counterstained with Eosin. Adobe Photoshop was used for final assembly of the images.

### Statistical Analysis

All values are presented as mean ± SEM. Two-way ANOVA was performed to compare the different parameters among the different groups. A significance level of *P *< 0.05 was assumed for all statistical evaluations. Statistics were computed with GraphPad Prism Software (San Diego, California).

## Results

### 1) Adenoviral mediated overexpression of GRK5-RH

#### a) Cell studies

**Figure 1:**
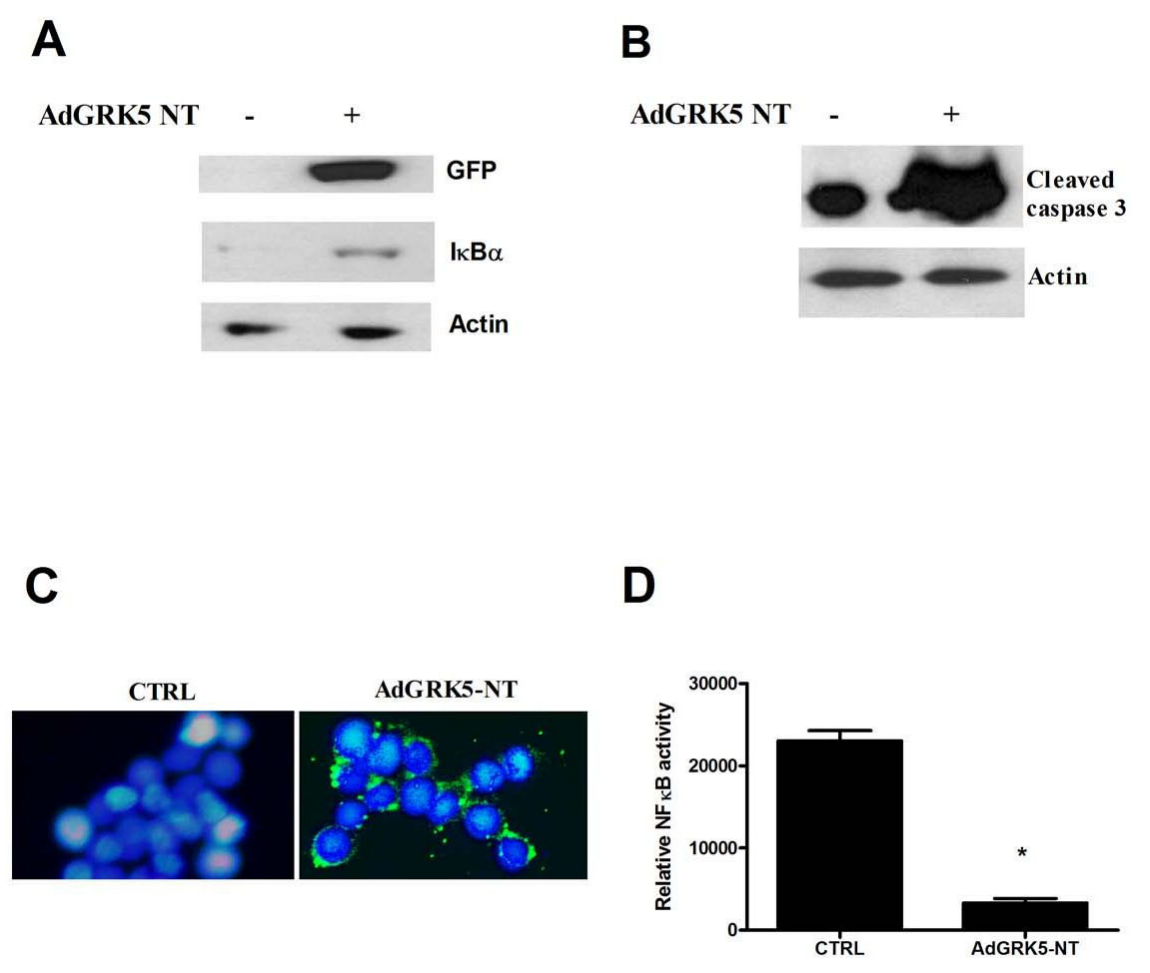
**Effects of GRK5-RH *in vitro *in KAT-4 cells overexpressed by adenovirus mediated gene transfer**. **A) **IκBα and GRK5-RH levels were analyzed in whole extracts by western blot, in KAT-4 overexpressing GRK5-RH by means of adenovirus mediated gene transfer. GRK5-RH increases IκBα levels. GRK5-RH expression was visualized by WB using anti-GFP antibody. **B) **To evaluate GRK5-RH effect on apoptosis, we analyzed the cleavage of caspase 3 by WB. The overexpression of GRK5-RH increases cleaved caspase 3 levels, suggesting that GRK5-RH causes an increase of apoptotic responses. **C) **This result was confirmed by Annexin V staining in fluorescence. GRK5-RH overexpression causes apoptosis as shown by Annexin-V staining compared to live cells (green = Annexin V; blue = nuclei) **D) **We evaluated GRK5 effects on NFκB activity by luciferase assay, in KAT-4 overexpressing GRK5-RH. Cells were transfected with plasmids coding for a κB-luciferase reporter (κB-Luc) and β-galactosidase (β-Gal) and lysates were analyzed by luciferase assay system (Promega). GRK5-RH inhibits NFκB transcriptional activity (*p < 0.05 vs control).

As previously demonstrated in endothelial cells [28], also in KAT-4 cells AdGRK5NT causes transgene expression that can be visualized through GFP fluorescence at green light (Figure 1A). This maneuver leads to IκBα accumulation in whole cell extracts (Figure 1A). In cancer cells NFκB activity inhibits apoptotic events. Thus, we assessed apoptosis in KAT-4 cells with adenoviral mediated overexpression of GRK5-RH. This maneuver increases cleaved caspase 3 levels compared to controls (Figure 1B). Similar results were achieved by Annexin V cell staining (Figure 1C). These responses associate with inhibition of NFκB activity, assessed by luciferase assay (Figure 1D). These data demonstrate that GRK5-RH inhibits NFκB transcriptional activity and biological effects also in cancer cells.

#### b) BALB/c nude mice tumors

In nude mice, the injection of 2 × 10^6 ^KAT-4 cells in the dorsal lateral region results in the development of a ~6 mm diameter tumor in 2 weeks, in about 70% of mice. Tumors were treated with different doses of AdGRK5-NT. One high dose of AdLac-Z was used as control.

**Figure 2:**
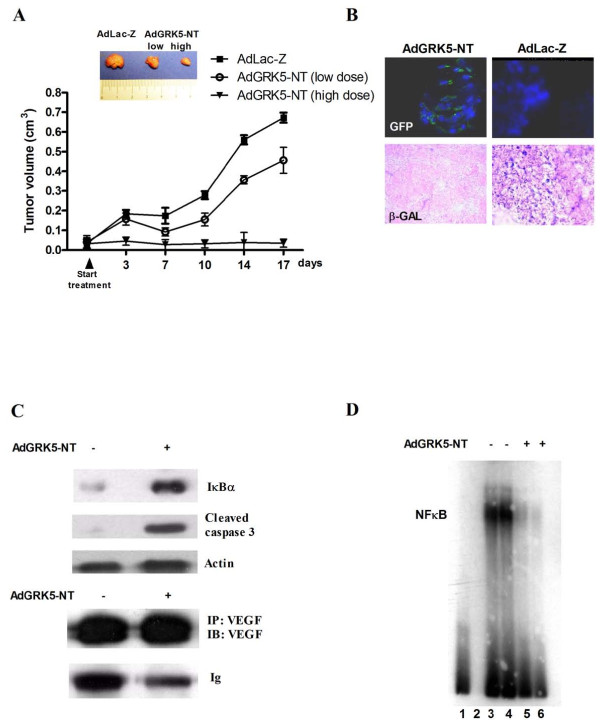
**Effects of GRK5-RH *in vivo *on tumor growth and biochemical analysis: treatment with adenovirus**. **A) **To validate our *in vitro *results, we studied the effects of adenoviral mediated overexpression of GRK5-RH in a cancer model *in vivo*. Tumor growth was measured twice a week by a caliper during all the treatment long (17 days of treatment). Low doses (10^8 ^pfu/ml in 200 ul) of AdGRK5-NT retard tumor growth compared to controls. High doses (10^11 ^pfu/ml in 200 μl) are more effective, inducing a complete inhibition of tumor growth. Figure also shows a representative image of tumors at the end of the treatment. **B) **17 days from starting treatment, mice were sacrified and tumors were taken for histological analysis. To ascertain GRK5-RH expression, cryostat sections were analyzed by direct observation of the green light at the fluorescence microscope. LAC-Z expression in control tumors was evaluated by X-GAL staining at direct light (blue staining indicates LAC-Z expression; eosin was used for counterstaining in red). **C) **Tumors were homogenized to analyze IκBα levels, VEGF production and apoptosis. Adenoviral mediated overexpression of GRK5-RH induces an increase of IκBα and cleaved caspase 3 levels and a reduction of VEGF expression in treated tumors compared to controls treated with AdLac-Z. **D) **Nuclear extracts from tumors were analyzed by EMSA. AdGRK5-NT inhibits NFκB activity compared to controls. Lane 1 = probe alone; Lane 2 = empty; Lane 3-4 = AdLac-Z different treated tumors; Lane 5-6 = AdGRK5-NT treated tumors.

Low doses of AdGRK5-NT retard tumor growth compared to controls (Figure 2A). High doses are more effective, inducing a complete inhibition of tumor growth (Figures 2A).

After 17 days of treatment, mice were sacrificed and tumors were taken for biochemical and histological analysis. Expression of AdGRK5-NT transgene is found by GFP fluorescence in the majority of cells within tumor when observed under green light (Figure 2B). In control tumors, LAC-Z expression is confirmed by blue X-GAL staining at direct light (Figure 2B). In order to evaluate the effect of GRK5-RH, we performed a western blot in homogenized tumors to analyze IκBα expression and apoptosis. Figure 2C shows that adenoviral mediated overexpression of GRK5-RH induces an increase of IκBα and cleaved caspase 3 levels in treated tumors compared to controls. NFκB also controls tumor angiogenesis. Therefore, we evaluated VEGF expression and found it significantly decreased in treated tumors compared to controls (Figure 2C). Results suggest that adenoviral mediated GRK5-RH expression inhibits NFκB activity in tumor cells. Indeed, EMSA analysis of tumors confirms NFκB inhibition: less NFκB is bound to genomic DNA in treated tumors compared to controls (Figure 2D).

### 2) Effects of administration of TAT-RH protein

#### a) Cell studies

**Figure 3:**
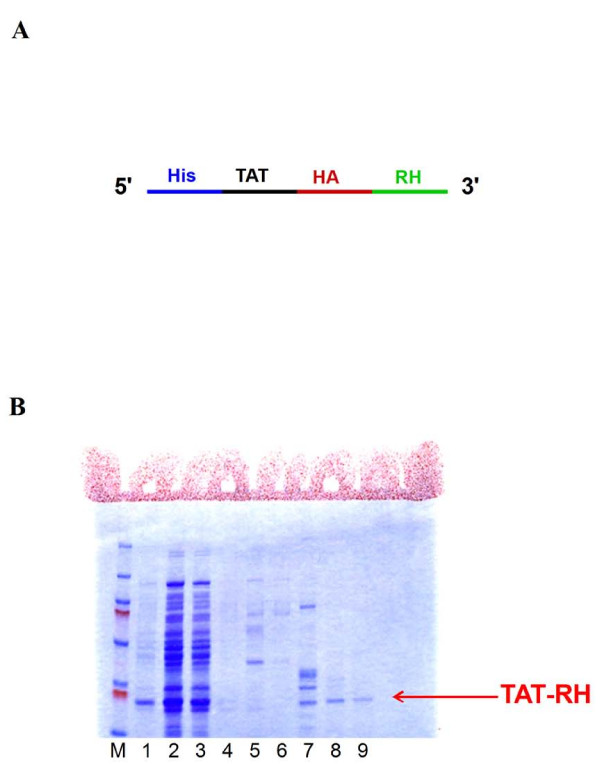
**TAT-RH protein design and purification**. **A) **We synthesized and purified a protein reproducing the RH domain of GRK5, TAT-RH. To this aim, RH gene was cloned into the pTAT-HA vector comprising the 11 aminoacid TAT domain, that is the minimum sequence of HIV TAT able to transduce into cells, and histidine and HA as N-terminal tags. **B) **The purified protein (~24 kDa) was visualized by electrophoretic analysis of samples from all steps of the purification process and gel staining. M = marker; Lane 1 = start; Lanes 2-3 = flow through; Lanes 4-6 = wash; Lanes 7-9 = imidazole.

To close up to a more pharmacological tool, we designed (Figure 3A) a protein (TAT-RH) reproducing the RH domain of GRK5, engineered to be actively transported into the cells through the retroviral TAT sequence and tested its anti-cancer property. Synthesis of the recombinant TAT-RH was performed in bacteria and column purification was confirmed by SDS-PAGE (Figure 3B).

**Figure 4:**
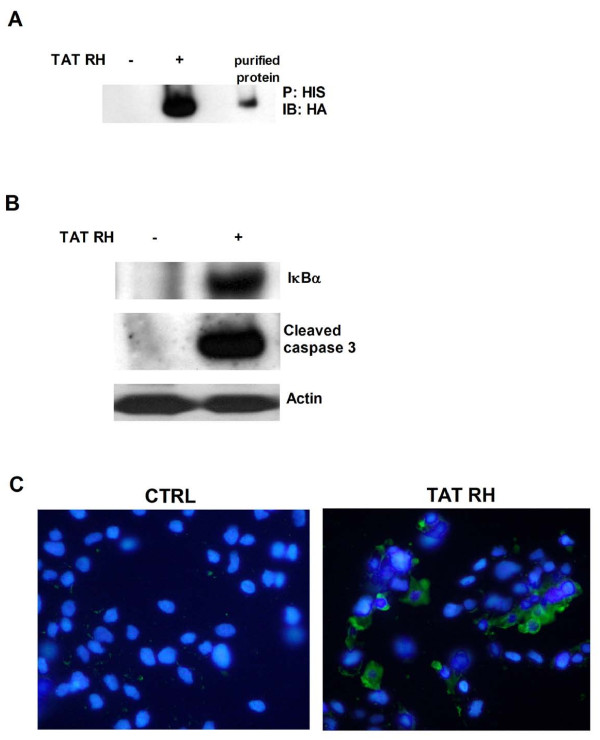
**Biological effects of TAT-RH in KAT-4 cells**. **A) **To evaluate the ability of the recombinant protein to autonomously enter into cells, we added TAT-RH (0,5 μg/ml) for 1 hour to culture medium and prepared cell lysates after three washes with saline buffer. We then evaluated the presence of TAT-RH in cell lysates by means of histidine precipitation and HA analysis by western blot. The purified protein was used as positive control. **B) **To verify the biological effect of TAT-RH, we evaluated IκBα and cleaved caspase 3 levels which were both increased by treatment. Actin was used as control. **C) **Apoptosis was also analized by Annexin V staining. TAT-RH increases Annexin V staining compared to controls (green = Annexin V; blue = nuclei).

**Figure 5:**
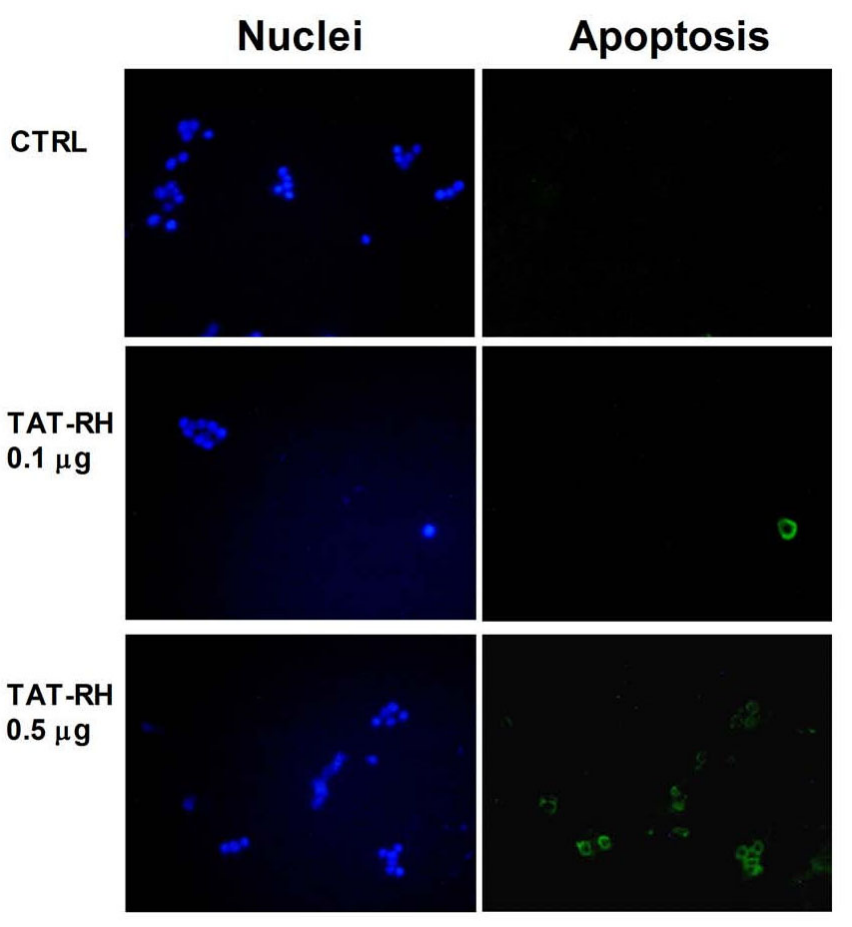
**Apoptosis analysis by Annexin V staining in TAT-RH treated cells**. To verify the effect of TAT-RH on NFκB-dependent apoptosis, we performed an Annexin V staining analysis in KAT-4 cells using different doses of TAT-RH to verify the minimum amount of protein needed to exert biological functions (green = Annexin V; blue = nuclei).

To verify the ability of TAT-RH protein to translocate into cells autonomously, we added the protein into the medium of cultured KAT-4 cells for 1 hour and verified its internalization by means of precipitation and western blot from cell lysates (Figure 4A). To verify the biological effect of TAT-RH, we evaluated IκBα and cleaved caspase 3 levels. TAT-RH increases both IκBα and cleaved caspase 3 levels (Figure 4B), thus confirming that our protein also in these cells causes apoptosis. Apoptosis was also assessed by Annexin V staining. Using different doses of TAT-RH, we found that the minimum amount of protein needed to exert biological functions is 0.5 μg/ml (Figure 5).

#### b) BALB/c nude mice tumors

**Figure 6:**
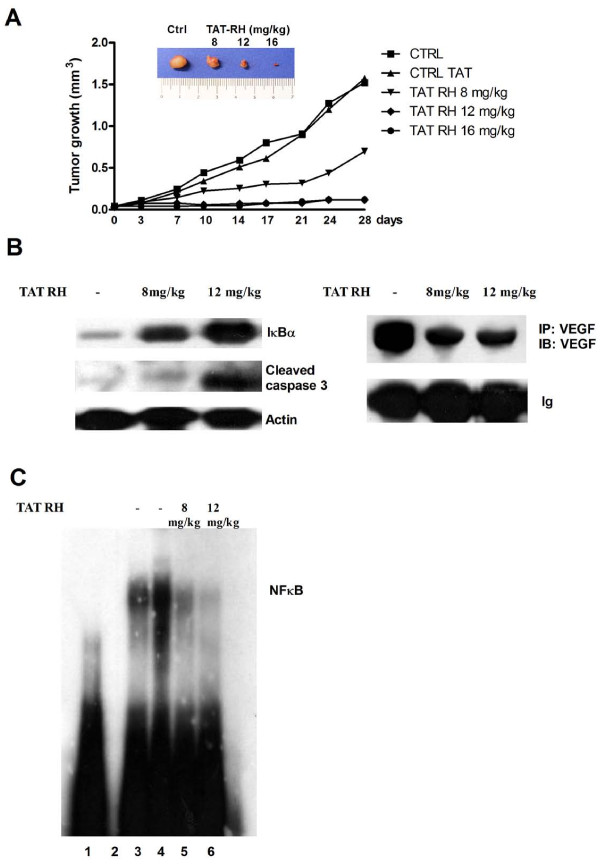
**Effects of TAT-RH *in vivo *on tumor growth and biochemical analysis**. **A) **We evaluated the effects of TAT-RH on tumor growth in BALB/c nude mice. High doses (16 mg/kg in 200 μl) lead to regression of tumors, intermediate doses (12 mg/kg in 200 μl) are able to completely inhibit tumor growth and low doses (8 mg/kg in 200 μl) can only delay tumor growth. Figure also shows a representative image of tumors at the end of the treatment (inset). **B) **Tumors were homogenized and the biological effects of TAT-RH treatment were evaluated by WB. TAT-RH leads to a dose-dependent increase of IκBα and cleaved caspase 3 and a reduction of VEGF levels. TAT treated tumors were used as controls. **C) **To evaluate the effect of TAT-RH on NFκB activity, we performed an EMSA using nuclear extracts from tumors. TAT-RH treatment reduces NFκB activity. Lane 1 = probe alone; Lane 2 = empty; Lane 3-4 = TAT treated tumors; Lane 5-6 = TAT-RH treated tumors.

We first tested the safety of *in vivo *administration of our protein in healthy nude mice. There were no significant changes in body weight among treated and control groups of mice indicating no toxicity of the treatment (Table 1). Internal organs (liver, lung, kidney) were then analyzed by histology. No morphological differences were found in treated mice compared to controls (data not shown). The effect of our protein on tumor growth appears to be dose-dependent. Indeed, high doses (16 mg/kg) lead to regression of tumors, intermediate doses (12 mg/kg) are able to completely inhibit tumor growth, since tumors maintain the same size of starting treatment, and low doses (8 mg/kg) can delay tumor growth (Figure 6A). As a control, we treated a group of 5 mice with the synthetic protein TAT lacking the GRK5-RH domain and a group with saline. Tumor was equal in mice treated with saline solution and those treated with TAT (Figure 6A).

**Table 1:** Mice body weight during TAT-RH treatment (gr)

Days of tretament	CTRL	TAT RH 16 mg/kg
**0**	26,2 ± 0,10	25,6 ± 0,28

**7**	25,8 ± 0,41	24,7 ± 0,23

**17**	22,7 ± 0,32	24,0 ± 0,35

**28**	22,7 ± 0,32	23,4 ± 0,35

We tested the safety of intraperitoneal administration of our protein in healthy nude mice. There were no significant changes in body weight among treated and control groups of mice indicating low toxicity of the treatment. Data are expressed as mean ± SE.

The effects of TAT-RH treatment on IκBα levels, apoptosis and angiogenesis, were assessed in control, 12 mg/kg and 8 mg/kg treated mice since at higher doses (16 mg/kg) of protein the tumor had a too small size for biochemical assessments. Figure 6B shows that TAT-RH treatment leads to a dose-dependent increase of IκBα and cleaved caspase 3 and a reduction of VEGF levels. Such effects is associated with NFκB activity inhibition, as evaluated by EMSA (Figure 6C).

**Figure 7:**
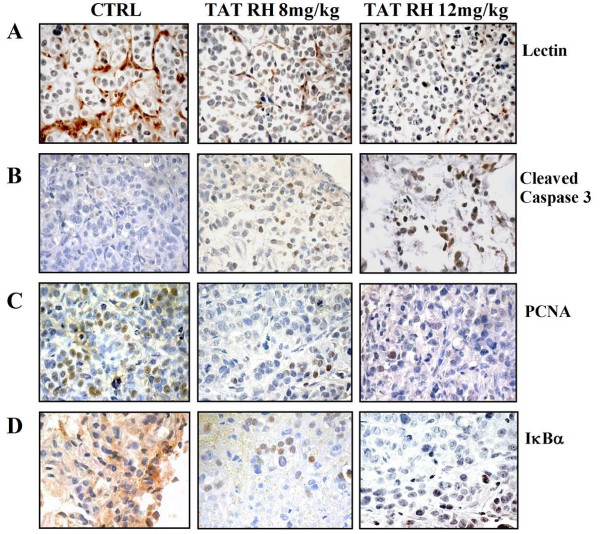
**Histological analysis of TAT-RH treated tumors**. Control and treated tumors were analized by histology. **A) **To evaluate angiogenesis in these tumors, lectin staining was used to visualize microvessels. TAT-RH reduces the formation of tumor vasculature. **B and C) **Cell death and proliferation were evaluated by analysis of cleaved caspase 3 and PCNA levels. TAT-RH treated tumors show increased cleaved caspase 3 levels (B) and reduced cell proliferation (C). **D) **We also evaluated IκBα subcellular localization. IκBα mainly localizes in the cytosol in control tumors while TAT-RH treatment causes its nuclear accumulation.

To measure angiogenesis in these tumors, lectin staining was used to visualize microvessels. Figure 7A shows that TAT-RH reduces the formation of tumor vasculature. Cell death and proliferation were evaluated by analysis of cleaved caspase 3 and PCNA levels. TAT-RH treated tumors show increased cleaved caspase 3 levels (Figure 7B) and reduced cell proliferation (Figure 7C). We also evaluated IκBα subcellular localization by immunohistochemistry. As previously demonstrated in endothelial cells, IκBα mainly localizes in the cytosol in control tumors while TAT-RH treatment causes its nuclear accumulation (Figure 7D).

**Figure 8:**
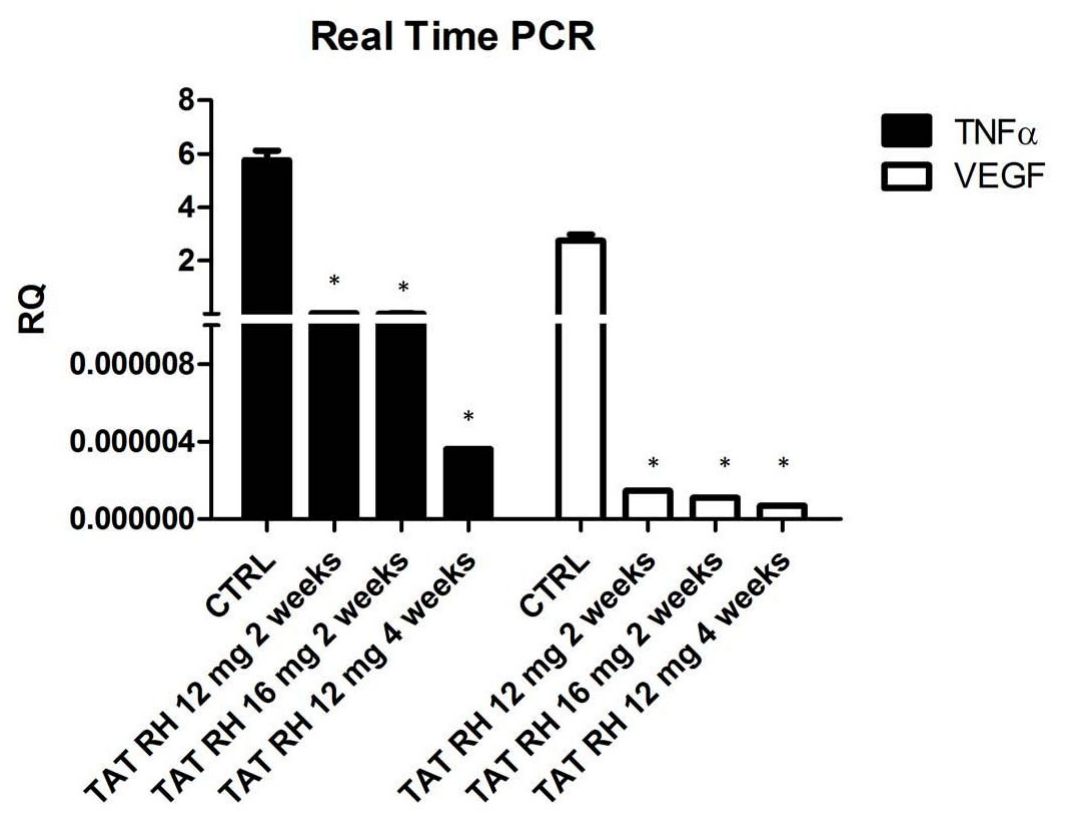
**Real-Time PCR analysis of inflammation and angiogenesis in TAT-RH treated tumors**. VEGF and TNFα expression was evaluated by Real Time PCR to assess the ability of TAT-RH to inhibit NFκB-dependent gene expression in cancer cells. At 2 weeks from starting treatment VEGF and TNFα expression is strongly inhibited in a dose dependent manner and prolonged treatments (4 weeks) increase such effect (*p < 0.05 vs control).

VEGF and TNFα expression was evaluated by Real Time PCR to assess the ability of TAT-RH to inhibit NFκB-dependent gene expression in cancer cells. Figure 8 shows that at 2 weeks from starting treatment both VEGF and TNFα expression is strongly inhibited and prolonged treatments (4 weeks) increase such effect.

## Discussion

The major finding of our manuscript is that GRK5-RH causes NFκB inhibition, through a novel mechanism of IκB stabilization, and leads to inhibition of growth and regression of size of tumors both *in vitro *and *in vivo*. We provide evidence using two sets of experiment: the first set, gathered through the overexpression of GRK5-RH by AdGRK5-RH, provides the proof of concept that GRK5-RH is able to inhibit cancer growth. It also represents the background for the synthesis and administration of a recombinant protein that resembles the inhibitory features of GRK5-RH, which was used in the second set of data, showing that TAT-RH can be used to obtain pharmacological inhibition of tumor growth.

It is known that a sustained, constitutive activation of NFκB contributes to malignant progression and therapeutic resistance in most of the major forms of human cancer, such as human lymphomas [31], carcinomas of the breast [32], prostate [33], lung [34], colon [22], pancreas [35], thyroid [21], head and neck [36] and cervix [37]. Thus, the modulation of NFκB activity represents an useful therapeutic strategy for cancer, since NFκB inhibition promotes apoptotic events induced by chemotherapy, reduces the high proliferative rate that characterizes tumor cells and inhibits metastasis [13]. We have recently demonstrated that the RH domain of GRK5 is an effective inhibitor of NFκB transcriptional activity in endothelial cells with implications in wound healing and tissue regeneration. In the present study we evaluated the effects of GRK5-RH in cancer. Our study was performed on an aggressive human carcinoma, the KAT-4 cell line. This tumor shows high proliferative rates and is NFkB sensitive [38]. Several strategies have been developed for blocking NFκB in tumors that include the inhibition of NFκB signaling pathway by proteasome inhibitors, IKK inhibitors, antioxidants or recombinant adenovirus-mediated overexpression of the IκBα gene, interfering with NFκB mRNA using specific anti-sense oligonucleotide [39-41]. We synthesized a protein reproducing the RH domain of GRK5, engineered to be actively transported into the cells by means of TAT domain without the support of other vehicles and tested its anti-cancer property. This approach has been already successfully used for protein transport in the treatment of several mouse model of cancer, inflammation and other diseases [42,43]. Our data suggest that TAT-RH protein enters into tumor cells, inhibits NFκB transcription activity and induces apoptosis, reduces tumor angiogenesis, blocks cell proliferation and consequently tumor growth in a dose dependent manner.

A clear advantage of our strategy of inhibition of NFκB is the fact that there is no overexpression of a transgene, but rather, the pharmacological inhibition of a mechanism of degradation of IκB which is both efficient and selective. One concern about inhibiting the NFκB pathway is the specificity. In particular, the proteasome which is responsible for IκB degradation has many other important functions. Thus, inhibition of proteasome activity could potentially cause severe side effects [44]. Since our strategy is based on the sterical interaction of TAT-RH and IκB and therefore does not require the inhibition of a general cellular mechanism, such as the proteasome, we hypothesize that the side effects of chronic treatment with TAT-RH, or a small molecule resembling it, will be of minimal intensity. Indeed, TAT-RH protein is not toxic when delivered through a systemic route, as it did not induce organ damage in mice. Thus, our data suggest that TAT-RH protein is a potent inhibitor of tumor growth both *in vitro *and *in vivo*, that is safe and well tolerated when administered systemically.

We believe that the anti-tumoral properties of GRK5-RH rely at least on two possible mechanisms. It is well known that apoptosis induced by chemotherapies is attenuated in tumor cells showing enhanced NFκB activity [1,45-47]. Here we demonstrated that GRK5-RH is able to induce apoptotic events in our model of cancer as evidenced by increased levels of the active form of caspase 3 (both by western blot and histological analysis) and Annexin V staining. Another feature that characterizes aggressive tumors is the ability to generate new vessels. Tumor angiogenesis has shown to be dependent on angiogenic factors, like chemokines and growth factors produced by macrophages, neutrophils and other inflammatory cells, all factors that have been shown to be regulated by NFκB [48,49]. GRK5-RH treatment reduces VEGF expression and production compared to controls and inhibits angiogenesis, thus providing another mechanism for retarding the *in vivo *growth of KAT-4 cells.

## Conclusion

This study demonstrates the ability of the RH domain of GRK5 to inhibit tumor growth through NFκB antagonism. This effect is achieved both *in vitro *and *in vivo *either through gene therapy or injection of TAT-RH protein. Thus, our data propose GRK5-RH as an useful therapeutic tool for cancer.

## Competing interests

The authors declare that they have no competing interests.

## Authors' contributions

GI designed research. DS, GS, AC, EL, and LP performed research. GI, DS and BT analyzed data and wrote the paper.

## References

[B1] Gilmore TD Introduction to NF-kappaB: players, pathways, perspectives Oncogene 2006 25 6680 6684 10.1038/sj.onc.1209954 17072321

[B2] Brasier AR The NF-kappaB regulatory network Cardiovasc Toxicol 2006 6 111 130 10.1385/CT:6:2:111 17303919

[B3] Perkins ND Integrating cell-signalling pathways with NF-kappaB and IKK function Nat Rev Mol Cell Biol 2007 8 49 62 10.1038/nrm2083 17183360

[B4] Gilmore TD The Rel/NF-kappaB signal transduction pathway: introduction Oncogene 1999 18 6842 6844 10.1038/sj.onc.1203237 10602459

[B5] Tian B Brasier AR Identification of a nuclear factor kappa B-dependent gene network Recent Prog Horm Res 2003 58 95 130 10.1210/rp.58.1.95 12795416

[B6] Jacobs MD Harrison SC Structure of an IkappaBalpha/NF-kappaB complex Cell 1998 95 749 758 10.1016/S0092-8674(00)81698-0 9865693

[B7] Baldwin AS Jr Series introduction: the transcription factor NF-kappaB and human disease J Clin Invest 2001 107 3 6 11134170 10.1172/JCI11891 PMC198555

[B8] Kumar A Takada Y Boriek AM Aggarwal BB Nuclear factor-kappaB: its role in health and disease J Mol Med 2004 82 434 448 10.1007/s00109-004-0555-y 15175863

[B9] Yamamoto Y Gaynor RB Role of the NF-kappaB pathway in the pathogenesis of human disease states Curr Mol Med 2001 1 287 296 10.2174/1566524013363816 11899077

[B10] Brown K Park S Kanno T Franzoso G Siebenlist U Mutual regulation of the transcriptional activator NF-kappa B and its inhibitor, I kappa B-alpha Proc Natl Acad Sci USA 1993 90 2532 2536 8460169 10.1073/pnas.90.6.2532 PMC46122

[B11] Shibata A Nagaya T Imai T Funahashi H Nakao A Seo H Inhibition of NF-kappaB activity decreases the VEGF mRNA expression in MDA-MB-231 breast cancer cells Breast Cancer Res Treat 2002 73 237 243 10.1023/A:1015872531675 12160329

[B12] Freund C Schmidt-Ullrich R Baurand A Dunger S Schneider W Loser P El-Jamali A Dietz R Scheidereit C Bergmann MW Requirement of nuclear factor-kappaB in angiotensin II- and isoproterenol-induced cardiac hypertrophy in vivo Circulation 2005 111 2319 2325 10.1161/01.CIR.0000164237.58200.5A 15870116

[B13] Ross JS Stagliano NE Donovan MJ Breitbart RE Ginsburg GS Atherosclerosis: a cancer of the blood vessels? Am J Clin Pathol 2001 116 Suppl S97 107 11993705 10.1309/YNCK-9R19-5JA3-K2K9

[B14] Frantz S Fraccarollo D Wagner H Behr TM Jung P Angermann CE Ertl G Bauersachs J Sustained activation of nuclear factor kappa B and activator protein 1 in chronic heart failure Cardiovasc Res 2003 57 749 756 10.1016/S0008-6363(02)00723-X 12618236

[B15] Karin M Cao Y Greten FR Li ZW NF-kappaB in cancer: from innocent bystander to major culprit Nat Rev Cancer 2002 2 301 310 10.1038/nrc780 12001991

[B16] Hanahan D Weinberg RA The hallmarks of cancer Cell 2000 100 57 70 10.1016/S0092-8674(00)81683-9 10647931

[B17] Aggarwal B Nuclear factor-kappaB: the enemy withincancer cell 2004 6 203 208 10.1016/j.ccr.2004.09.003 15380510

[B18] Shattuck-Brandt RL Richmond A Enhanced degradation of I-kappaB alpha contributes to endogenous activation of NF-kappaB in Hs294T melanoma cells Cancer Res 1997 57 3032 3039 9230219

[B19] Amiri KI Richmond A Role of nuclear factor-kappa B in melanoma Cancer Metastasis Rev 2005 24 301 313 15986139 10.1007/s10555-005-1579-7 PMC2668255

[B20] Visconti R Cerutti J Battista S Fedele M Trapasso F Zeki K Miano MP de Nigris F Casalino L Curcio F Expression of the neoplastic phenotype by human thyroid carcinoma cell lines requires NFkappaB p65 protein expression Oncogene 1997 15 1987 1994 10.1038/sj.onc.1201373 9365245

[B21] Pacifico F Mauro C Barone C Crescenzi E Mellone S Monaco M Chiappetta G Terrazzano G Liguoro D Vito P Oncogenic and anti-apoptotic activity of NF-kappa B in human thyroid carcinomas J Biol Chem 2004 279 54610 54619 10.1074/jbc.M403492200 15475567

[B22] Kojima M Morisaki T Sasaki N Nakano K Mibu R Tanaka M Katano M Increased nuclear factor-kB activation in human colorectal carcinoma and its correlation with tumor progression Anticancer Res 2004 24 675 681 15161011

[B23] Kane RC Bross PF Farrell AT Pazdur R Velcade: U.S. FDA approval for the treatment of multiple myeloma progressing on prior therapy Oncologist 2003 8 508 513 10.1634/theoncologist.8-6-508 14657528

[B24] Jimi E Aoki K Saito H D'Acquisto F May MJ Nakamura I Sudo T Kojima T Okamoto F Fukushima H Selective inhibition of NF-kappa B blocks osteoclastogenesis and prevents inflammatory bone destruction in vivo Nat Med 2004 10 617 624 10.1038/nm1054 15156202

[B25] Dewan MZ Terashima K Taruishi M Hasegawa H Ito M Tanaka Y Mori N Sata T Koyanagi Y Maeda M Rapid tumor formation of human T-cell leukemia virus type 1-infected cell lines in novel NOD-SCID/gammac(null) mice: suppression by an inhibitor against NF-kappaB J Virol 2003 77 5286 5294 12692230 10.1128/JVI.77.9.5286-5294.2003 PMC153944

[B26] Van Antwerp DJ Martin SJ Kafri T Green DR Verma IM Suppression of TNF-alpha-induced apoptosis by NF-kappaB Science 1996 274 787 789 10.1126/science.274.5288.787 8864120

[B27] Wang CY Mayo MW Baldwin AS Jr TNF- and cancer therapy-induced apoptosis: potentiation by inhibition of NF-kappaB Science 1996 274 784 787 10.1126/science.274.5288.784 8864119

[B28] Sorriento D Ciccarelli M Santulli G Campanile A Altobelli GG Cimini V Galasso G Astone D Piscione F Pastore L The G-protein-coupled receptor kinase 5 inhibits NFkappaB transcriptional activity by inducing nuclear accumulation of IkappaB alpha Proc Natl Acad Sci USA 2008 105 17818 17823 19008357 10.1073/pnas.0804446105 PMC2584738

[B29] Schweppe RE Klopper JP Korch C Pugazhenthi U Benezra M Knauf JA Fagin JA Marlow LA Copland JA Smallridge RC Haugen BR Deoxyribonucleic acid profiling analysis of 40 human thyroid cancer cell lines reveals cross-contamination resulting in cell line redundancy and misidentification J Clin Endocrinol Metab 2008 93 4331 4341 18713817 10.1210/jc.2008-1102 PMC2582569

[B30] Becker-Hapak M McAllister SS Dowdy SF TAT-mediated protein transduction into mammalian cells Methods 2001 24 247 256 10.1006/meth.2001.1186 11403574

[B31] Bargou RC Emmerich F Krappmann D Bommert K Mapara MY Arnold W Royer HD Grinstein E Greiner A Scheidereit C Dorken B Constitutive nuclear factor-kappaB-RelA activation is required for proliferation and survival of Hodgkin's disease tumor cells J Clin Invest 1997 100 2961 2969 9399941 10.1172/JCI119849 PMC508507

[B32] Sovak MA Bellas RE Kim DW Zanieski GJ Rogers AE Traish AM Sonenshein GE Aberrant nuclear factor-kappaB/Rel expression and the pathogenesis of breast cancer J Clin Invest 1997 100 2952 2960 9399940 10.1172/JCI119848 PMC508506

[B33] Suh J Payvandi F Edelstein LC Amenta PS Zong WX Gelinas C Rabson AB Mechanisms of constitutive NF-kappaB activation in human prostate cancer cells Prostate 2002 52 183 200 10.1002/pros.10082 12111695

[B34] Mukhopadhyay T Roth JA Maxwell SA Altered expression of the p50 subunit of the NF-kappa B transcription factor complex in non-small cell lung carcinoma Oncogene 1995 11 999 1003 7675461

[B35] Wang W Abbruzzese JL Evans DB Larry L Cleary KR Chiao PJ The nuclear factor-kappa B RelA transcription factor is constitutively activated in human pancreatic adenocarcinoma cells Clin Cancer Res 1999 5 119 127 9918209

[B36] Ondrey FG Dong G Sunwoo J Chen Z Wolf JS Crowl-Bancroft CV Mukaida N Van Waes C Constitutive activation of transcription factors NF-(kappa)B, AP-1, and NF-IL6 in human head and neck squamous cell carcinoma cell lines that express pro-inflammatory and pro-angiogenic cytokines Mol Carcinog 1999 26 119 129 10.1002/(SICI)1098-2744(199910)26:2<119::AID-MC6>3.0.CO;2-N 10506755

[B37] Nair A Venkatraman M Maliekal TT Nair B Karunagaran D NF-kappaB is constitutively activated in high-grade squamous intraepithelial lesions and squamous cell carcinomas of the human uterine cervix Oncogene 2003 22 50 58 10.1038/sj.onc.1206043 12527907

[B38] Demeter JG De Jong SA Lawrence AM Paloyan E Anaplastic thyroid carcinoma: risk factors and outcome Surgery 1991 110 956 961 1745983

[B39] Cho S Urata Y Iida T Goto S Yamaguchi M Sumikawa K Kondo T Glutathione downregulates the phosphorylation of I kappa B: autoloop regulation of the NF-kappa B-mediated expression of NF-kappa B subunits by TNF-alpha in mouse vascular endothelial cells Biochem Biophys Res Commun 1998 253 104 108 10.1006/bbrc.1998.9697 9875227

[B40] Schreck R Meier B Mannel DN Droge W Baeuerle PA Dithiocarbamates as potent inhibitors of nuclear factor kappa B activation in intact cells J Exp Med 1992 175 1181 1194 1314883 10.1084/jem.175.5.1181 PMC2119220

[B41] Higgins KA Perez JR Coleman TA Dorshkind K McComas WA Sarmiento UM Rosen CA Narayanan R Antisense inhibition of the p65 subunit of NF-kappa B blocks tumorigenicity and causes tumor regression Proc Natl Acad Sci USA 1993 90 9901 9905 8234333 10.1073/pnas.90.21.9901 PMC47680

[B42] Snyder EL Dowdy SF Recent advances in the use of protein transduction domains for the delivery of peptides, proteins and nucleic acids in vivo Expert Opin Drug Deliv 2005 2 43 51 10.1517/17425247.2.1.43 16296734

[B43] Wadia JS Dowdy SF Transmembrane delivery of protein and peptide drugs by TAT-mediated transduction in the treatment of cancer Adv Drug Deliv Rev 2005 57 579 596 10.1016/j.addr.2004.10.005 15722165

[B44] Yamamoto Y Gaynor RB Therapeutic potential of inhibition of the NF-kappaB pathway in the treatment of inflammation and cancer J Clin Invest 2001 107 135 142 11160126 10.1172/JCI11914 PMC199180

[B45] Arlt A Gehrz A Muerkoster S Vorndamm J Kruse ML Folsch UR Schafer H Role of NF-kappaB and Akt/PI3K in the resistance of pancreatic carcinoma cell lines against gemcitabine-induced cell death Oncogene 2003 22 3243 3251 10.1038/sj.onc.1206390 12761494

[B46] Brach MA Hass R Sherman ML Gunji H Weichselbaum R Kufe D Ionizing radiation induces expression and binding activity of the nuclear factor kappa B J Clin Invest 1991 88 691 695 1864978 10.1172/JCI115354 PMC295415

[B47] Hwang S Ding A Activation of NF-kappa B in murine macrophages by taxol Cancer Biochem Biophys 1995 14 265 272 7767900

[B48] Huang S DeGuzman A Bucana CD Fidler IJ Nuclear factor-kappaB activity correlates with growth, angiogenesis, and metastasis of human melanoma cells in nude mice Clin Cancer Res 2000 6 2573 2581 10873114

[B49] Chilov D Kukk E Taira S Jeltsch M Kaukonen J Palotie A Joukov V Alitalo K Genomic organization of human and mouse genes for vascular endothelial growth factor C J Biol Chem 1997 272 25176 25183 10.1074/jbc.272.40.25176 9312130

